# Supplementary material for: Design and Testing of Effective Primers for Amplification of the orf7 Gene of Phage WO Associated with Andricus hakonensis
Source: Insects. 2021 Aug 9;12(8):713. doi: 10.3390/insects12080713 (PMC8397071; doi:10.3390/insects12080713)
Supplement: Supplementary file 1 [file insects-12-00713-s001.zip › TABLE S1 .pdf]

Supplementary **Table S1.** Genomes used for primer design in this study

Supergroup	Strain	Host	Accession
A	*w*Mel	*Drosophila melanogaster*	NC002978
A	*w*Inc_Cu	*Drosophila incompta*	CP011148
A	*w*Ri	*Drosophila simulans*	CP001391
A	*w*CauA	*Carposina sasakii*	CP041215
A	*w*Ha	*Drosophila simulans*	NC021089
A	*w*MelPop	*Drosophila melanogaster*	NZ_AQQE00000000.1
A	*w*Yak	*Drosophila yakuba*	NZ_VCEF00000000.1
A	*w*Au	*Drosophila simulans*	NZ_LK055284.1
A	*w*Rec	*Drosophila recens*	NZ_JQAM00000000.1
A	*w*Ana	*Drosophila ananassae*	NZ_CP042904.1
A	*w*Spc	*Drosophila subpulchrella*	NZ_NTHL00000000.1
A	*w*Suzi	*Drosophila suzukii*	NZ_CAOU00000000.2
A	*w*OneA1	*Nasonia oneida*	NZ_QESS00000000.1
A	*w*Uni	*Muscidifurax uniraptor*	NZ_MUJL01000001.1
A	*w*VitA	*Nasonia vitripennis*	NZ_MUJM01000001.1
A	*w*Gmo	*Glossina morsitans*	NZ_AWUH00000000.1
A	*w*Nfla	*Nomada flava*	NZ_LYUW00000000.1
A	*w*Nleu	*Nomada leucophthalma*	NZ_LYUV00000000.1
A	*w*Npa	*Nomada panzeri*	NZ_LYUX00000000.1
A	*w*Nfe	*Nomada ferruginata*	NZ_LYUY00000000.1
A	*w*DacA	*Dactylopius coccus*	NZ_LSYX00000000.1
B	*w*Meg	*Chrysomya megacephala*	CP021120
B	*w*PipPel	*Culex quinquefasciatus Pel*	NC010981
B	*w*Tpre	*Trichogramma pretiosum*	CM003641
B	*w*AlbB	*Aedes albopictus*	CP031221
B	*w*Bol1-b	*Hypolimnas bolina*	NZ_CAOH00000000.1
B	*w*PipMol	*Culex molestus*	NZ_CTEH00000000.1
B	*w*Aus	*Plutella Australiana*	NZ_MRWX01000001
B	*w*Ob	*Operophtera brumata*	NZ_JYPC00000000.1
B	*w*Lcla	*Leptopilina clavipes*	NZ_QJHA00000000.1
B	*w*VitB	*Nasonia vitripennis*	NZ_AERW00000000.1
B	*w*DacB	*Dactylopius coccus*	NZ_LSYY00000000.1
B	*w*Stri	*Laodelphax striatellus*	NZ_MUIX00000000.1
B	*w*Di	*Diaphorina citri*	NZ_AMZJ01000001.1
B	*w*Lug	*Nilaparvata lugens*	NZ_MUIY00000000.1
B	*w*Con	*Cylisticus convexus*	NZ_QPIP00000000.1
B	*w*VulC	*Armadillidium vulgare*	NZ_ALWU01000001.1
