# Supplementary figures and images for: Design and Testing of Effective Primers for Amplification of the orf7 Gene of Phage WO Associated with Andricus hakonensis
Source: Insects. 2021 Aug 9;12(8):713. doi: 10.3390/insects12080713 (PMC8397071; doi:10.3390/insects12080713)

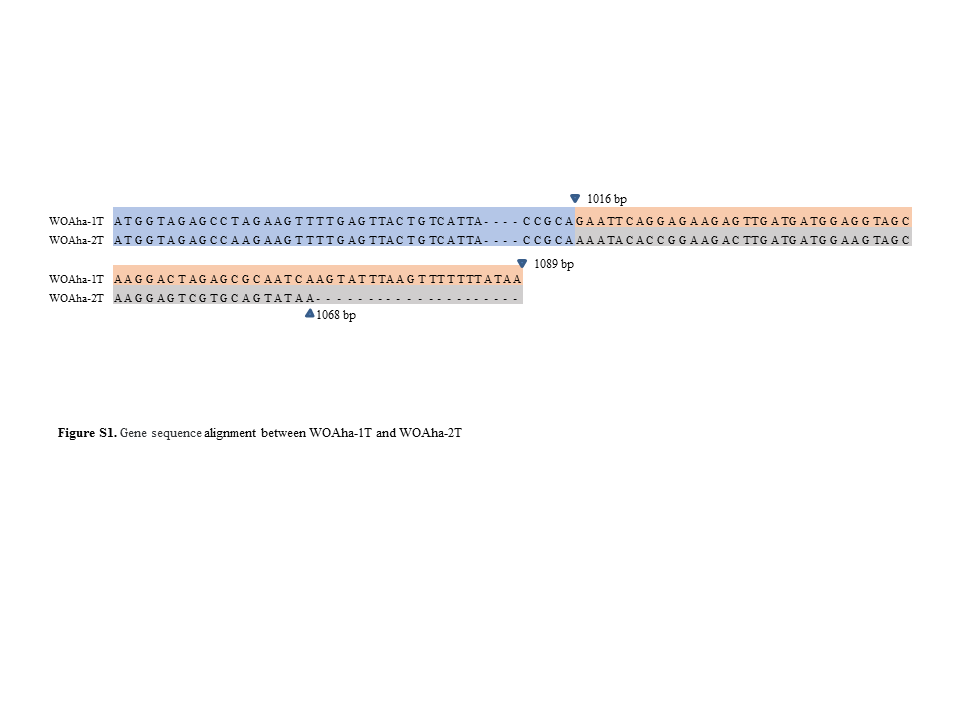

Supplement: Supplementary file 1 [file insects-12-00713-s001.zip › Fig S1-S4/fig S1 .tif]

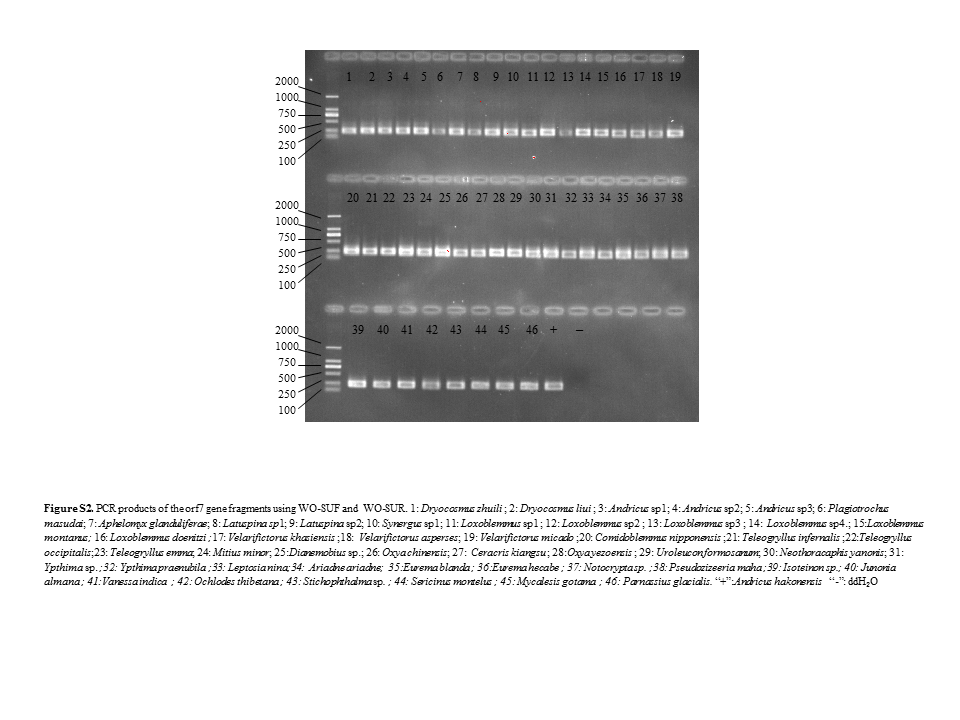

Supplement: Supplementary file 1 [file insects-12-00713-s001.zip › Fig S1-S4/fig S2 .TIF]

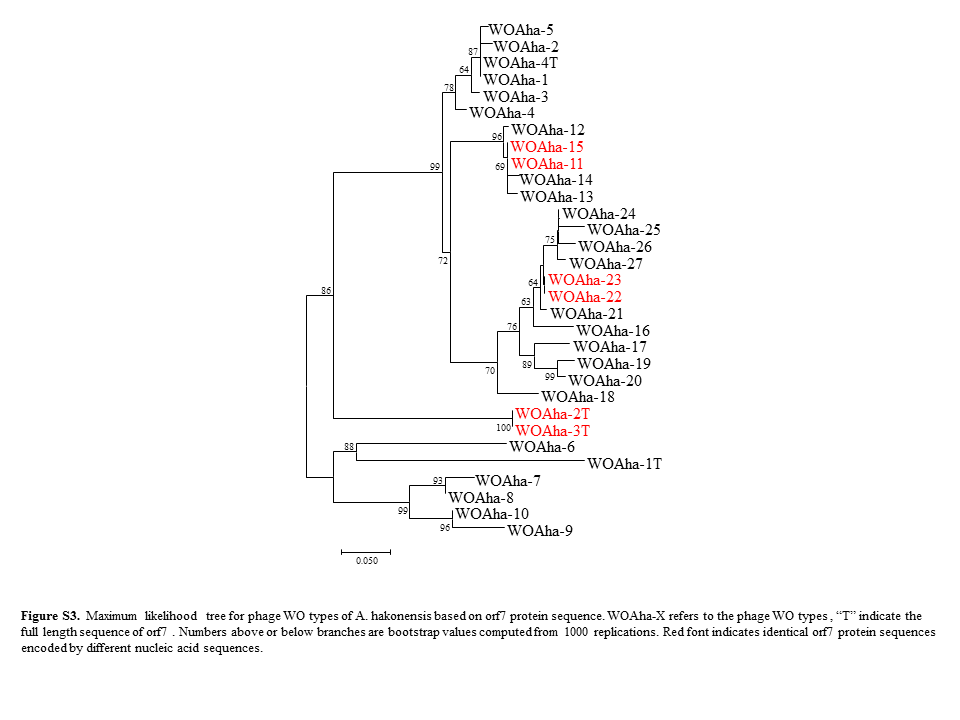

Supplement: Supplementary file 1 [file insects-12-00713-s001.zip › Fig S1-S4/fig S3 .TIF]

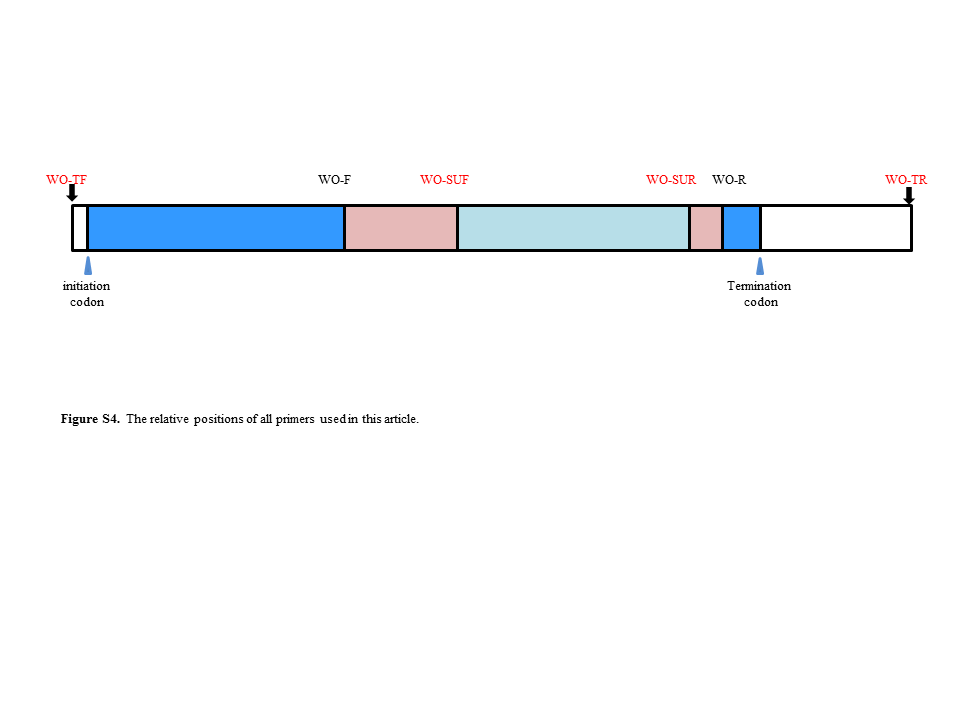

Supplement: Supplementary file 1 [file insects-12-00713-s001.zip › Fig S1-S4/Fig S4.tif]
